# In-Situ Growth of NiAl-Layered Double Hydroxide on AZ31 Mg Alloy towards Enhanced Corrosion Protection

**DOI:** 10.3390/nano8060411

**Published:** 2018-06-07

**Authors:** Xin Ye, Zimin Jiang, Linxin Li, Zhi-Hui Xie

**Affiliations:** 1 Chemical Synthesis and Pollution Control Key Laboratory of Sichuan Province, China West Normal University, Nanchong 637002, China; xinchem4@163.com (X.Y.); Linxinyj@163.com (L.L.); 2 College of Foreign Language Education, China West Normal University, Nanchong 637002, China; jenny8116@126.com

**Keywords:** Mg alloy, LDH, corrosion, deposition, coating

## Abstract

NiAl-layered double hydroxide (NiAl-LDH) coatings grown in-situ on AZ31 Mg alloy were prepared for the first time utilizing a facile hydrothermal method. The surface morphologies, structures, and compositions of the NiAl-LDH coatings were characterized by scanning electron microscopy (SEM), three dimensional (3D) optical profilometer, X-ray diffractometer (XRD), Fourier transform infrared spectrometer (FT-IR), and X-ray photoelectron spectroscopy (XPS). The results show that NiAl-LDH coating could be successfully deposited on Mg alloy substrate using different nickel salts, i.e., carbonate, nitrate, and sulfate salts. Different coatings exhibit different surface morphologies, but all of which exhibit remarkable enhancement in corrosion protection in 3.5 wt % NaCl corrosive electrolyte. When nickel nitrate was employed especially, an extremely large impedance modulus at a low frequency of 0.1 Hz (|*Z*|*_f_*_= 0.1 Hz_), 11.6 MΩ cm^2^, and a significant low corrosion current density (*j*_corr_) down to 1.06 nA cm^−2^ are achieved, demonstrating NiAl-LDH coating’s great potential application in harsh reaction conditions, particularly in a marine environment. The best corrosion inhibition of NiAl-LDH/CT coating deposited by carbonate may partially ascribed to the uniform and vertical orientation of the nanosheets in the coating.

## 1. Introduction

Mg alloy has excellent physical and mechanical properties but a high chemical reactivity and susceptibility to corrosion, which hinders its practical application and development in more fields [1]. Efforts by formation of protective coatings on Mg alloy surface to decrease the corrosion rate and extend the serving time have aroused an increasing interest in the area of surface engineering [1,2]. Many traditional coating methods are adopted to protect Mg alloy from corrosion, such as electroless Ni plating [3,4]. However, more and more attendant problems, especially environmental issues, emerge with increasing usage of these techniques, such as the high cost and complexity in dealing with the waste plating bath, and the detrimental effect of Cr to the ecosystem [3]. Thus, it is highly urgent to find state-of-the-art alternative coatings with low pollution emissions but with efficient corrosion inhibition.

Layered double hydroxides (LDHs) have received extensive attention for potential application in supercapacitor [5], catalysis [6], adsorbents [7], and corrosion protection [8] because of their diversity in compositions and structures. The charge of the metal layer in the LDHs is compensated by interlayer anions such as CO_3_^2^^−^. Once CO_3_^2^^−^ anions are intercalated, it cannot be exchanged by most corrosive anions, such as SO_4_^2^^−^ and Cl^−^, due to the high ion-exchange equilibrium constant of CO_3_^2^^−^ anions, thereby the corrosion of the substrate under the LDHs coating will be delayed. From this point of view, CO_3_^2^^−^ anions intercalated LDHs are an ideal choice for obtaining a LDHs coating with high corrosion inhibition capacity. The most widely reported CO_3_^2^^−^ intercalated LDHs on Mg alloy matrix is MgAl-LDHs coating, which is obtained, in most cases, by in-situ growth technology owing to the simplification of operation and strong adhesion of the coating [9]. Recently, MgFe-LDH and MgCr-LDH films were also obtained on anodized AZ31 Mg alloy by in-situ growth measurement [10]. However, the orders of magnitude of the *j*_corr_ of these in-situ grown LDH coatings are higher than −8, mostly range from −5 to −7 (Table 1) [8,11,12,13,14]. To break the bottleneck of *j*_corr_, it is advisable to prepare carbonate-based LDHs film by attempting more different divalent or trivalent metallic cations. Though most recent research results proved that NiAl-LDH nanoparticles possess good photocatalytic performance [15], in-situ growth of NiAl-LDH film on Mg alloy for corrosion protection has not yet been reported to date.

**Figure 1:**
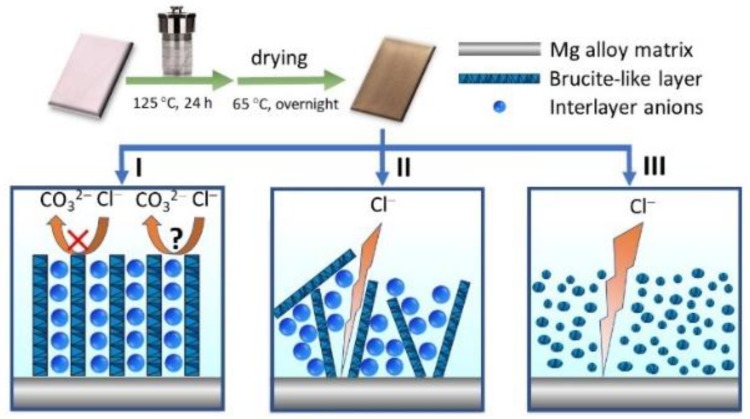
Schematic illustration of various microstructures of NiAl-LDH coatings prepared with different nickel salts. (I) nickel carbonate; (II) nickel nitrate; and (III) nickel sulfate. The interlayer anions are not given in case III.

Herein, we report a facile hydrothermal measurement to in-situ growth of NiAl-LDH nanocomposite on Mg alloy by use of alkaline solutions with three different nickel salts for preparation of a highly enhanced corrosion-resistant coating with an extraordinary low corrosion rate (Figure 1). The NiAl-LDH coating prepared by nickel carbonate exhibits uniformly and vertically aligned nanoarrays with an extremely large impedance modulus at a low frequency of 0.1 Hz (|*Z*|*_f_*_= 0.1 Hz_), 11.6 MΩ cm^2^, and a significantly low *j*_corr_ down to 1.06 nA cm^−2^ in 3.5 wt % NaCl corrosive electrolyte, which outperforms the values of the foregoing achieved LDH coating on Mg alloy (Table 1) [8,10,13,14,16,17,18]. The NiAl-LDH coatings were characterized, and the enhanced corrosion inhibition mechanism was proposed and discussed.

## 2. Methods

### 2.1. Materials and Reagents

The matrix used is AZ31 Mg alloy with a chemical composition in wt %: 2.75 Al, 1.15 Zn, 0.16 Mn, and Mg balance. Primary chemicals include nickel carbonate (AR), nickel nitrate (≥98%), nickel sulphate (≥98.5%), sodium carbonate (≥99.8%), and sodium hydroxide (≥98%). Chemicals were acquired from Aladdin Industrial Inc. (Shanghai, China) and Sinopharm Chemical Reagent Co., Ltd. (Shanghai, China), and all chemicals were used without further purification. Ultrapure water used in the experiments was obtained using a water purification system (UPT-II-10T, Ulupure Corporation, Chengdu, China) with a resistivity of 18.2 MΩ cm at 25 °C.

### 2.2. Preparation of NiAl-LDH Coatings on Mg Alloy

All the NiAl-LDH coatings were grown on Mg alloy substrate by a simple one step hydrothermal method with the same steps and conditions but with three different nickel salts. The obtained coatings by use of nickel carbonate, nickel nitrate, and nickel sulphate are denoted as NiAl-LDH/CT, NiAl-LDH/NT, and NiAl-LDH/ST coatings, respectively. The mole ratio of Ni^2+^, Al^3+^, and CO_3_^2−^ in the precursor solution is 6:2:1. Take the preparation of NiAl-LDH/CT coating, for example, which is described as follows:

*Pre-treatment of substrate*: The AZ31 Mg alloy was ground mechanically with SiC waterproof sand paper and degreased in an alkaline solution at 65 °C for 10 min [3].

*Preparation of precursor solution*: 40 mL aluminum nitrate solution (0.002 mol Al(NO_3_)_3_·9H_2_O) was first added to a nickel solution (~40 mL) containing 0.002 mol NiCO_3·_2Ni(OH)_2_·4H_2_O. Then, 0.001 mol anhydrous Na_2_CO_3_ was added, followed by pH adjustment of the solution to about 12 by use of a NaOH solution (~3.7 mL). Finally, the above solution was diluted to a volume of 100 mL by adding ultrapure water.

*Growth of NiAl-LDH film*: The solution above was then transferred into a 100 mL Teflon-lined autoclave where a pre-treated Mg alloy has been placed. Then, the autoclave was placed and kept in an oven with a temperature of 125 °C for 24 h for in-situ growth of NiAl-LDH films on the matrix.

After that, the samples were taken out of the autoclave, rinsed with water, dried overnight in an oven at 65 °C, and designed as NiAl-LDH/CT coating. The amount of nickel salts is 0.006 mol for deposition of NiAl-LDH/NT and NiAl-LDH/ST coatings, which is the sole difference in comparison with preparation of NiAl-LDH/CT coating.

### 2.3. Characterization and Electrochemical Tests

The surface morphologies and roughness of the different NiAl-LDH coatings were observed by scanning electron microscopy (SEM, Hitachi S-4800, Hitachi High-Technologies Corporation, Tokyo, Japan), and three dimensional (3D) optical profilometer (Bruker Contour GT-K, Billerica, MA, USA), respectively. The arithmetical mean deviation of the profile (*R*_a_) is used to estimate the roughness. The microstructures of the coatings were identified by X-ray diffractometer (XRD, D8 Advance). Fourier transform infrared spectroscopic acquisition of the specimens were obtained (FT-IR, Nicolet 6700, Thermo Scientific, Waltham, MA, USA) in the range of 4000~500 cm^−1^. The elemental compositions of the sample surface were recorded by X-ray photoelectron spectroscopy (XPS, 250Xi, Thermo Scientific, Waltham, MA, USA) and energy dispersive X-ray spectroscopy (EDS) equipped in SEM. The water contact angles were measured with a water drop volume of 5 μL, utilizing optical contact angle meter (JC2000D, Shanghai Zhongchen Digital Technology Apparatus Co., Ltd., Shanghai, China) at 298 K. The electrochemical impedance spectroscopy (EIS) and Tafel curves were achieved using a classical three-electrode system with saturated calomel electrode (SCE) as reference electrode, Pt foil as counter electrode, and freshly ground bare Mg alloy and as-prepared NiAl-LDH coatings as working electrode (exposed area: 1 × 1 cm^2^) on an electrochemical workstation (CHI660E, Chenhua, Shanghai, China). The EIS frequencies range from 10^5^~0.1 Hz using an AC perturbation of 10 mV versus the open circuit potential (OCP). The Tafel measurement was performed at a scan rate of 5 mV/s in the potential region of ±500 mV versus OCP. All the electrochemical tests were carried out in a 3.5 wt % NaCl solution at 298 K.

## 3. Results and Discussion

**Figure 2:**
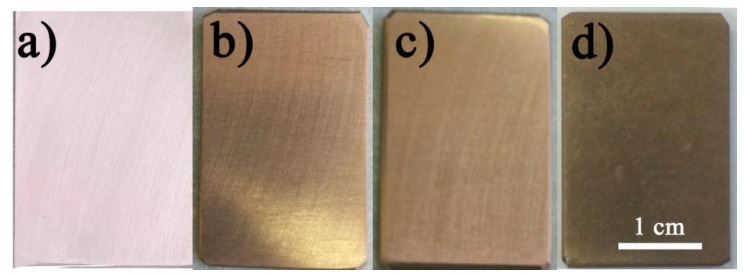
Digital photos of (**a**) bare Mg alloy; (**b**) NiAl-LDH/CT; (**c**) NiAl-LDH/NT; and (**d**) NiAl-LDH/ST coatings.

The digital photos of bare Mg alloy and three different NiAl-LDH coatings are shown in Figure 2. The substrate shows a shiny surface after polishing and pre-treatment processes, as shown in Figure 2a. After coating, all the samples show a light bronze-like surface in color, suggesting the successfully deposition of NiAl-LDH coating on the Mg alloy (Figure 2b–d). The surface brightness of NiAl-LDH/CT and NiAl-LDH/NT coatings are very close, and both of which are brighter than that of NiAl-LDH/ST coating.

**Figure 3:**
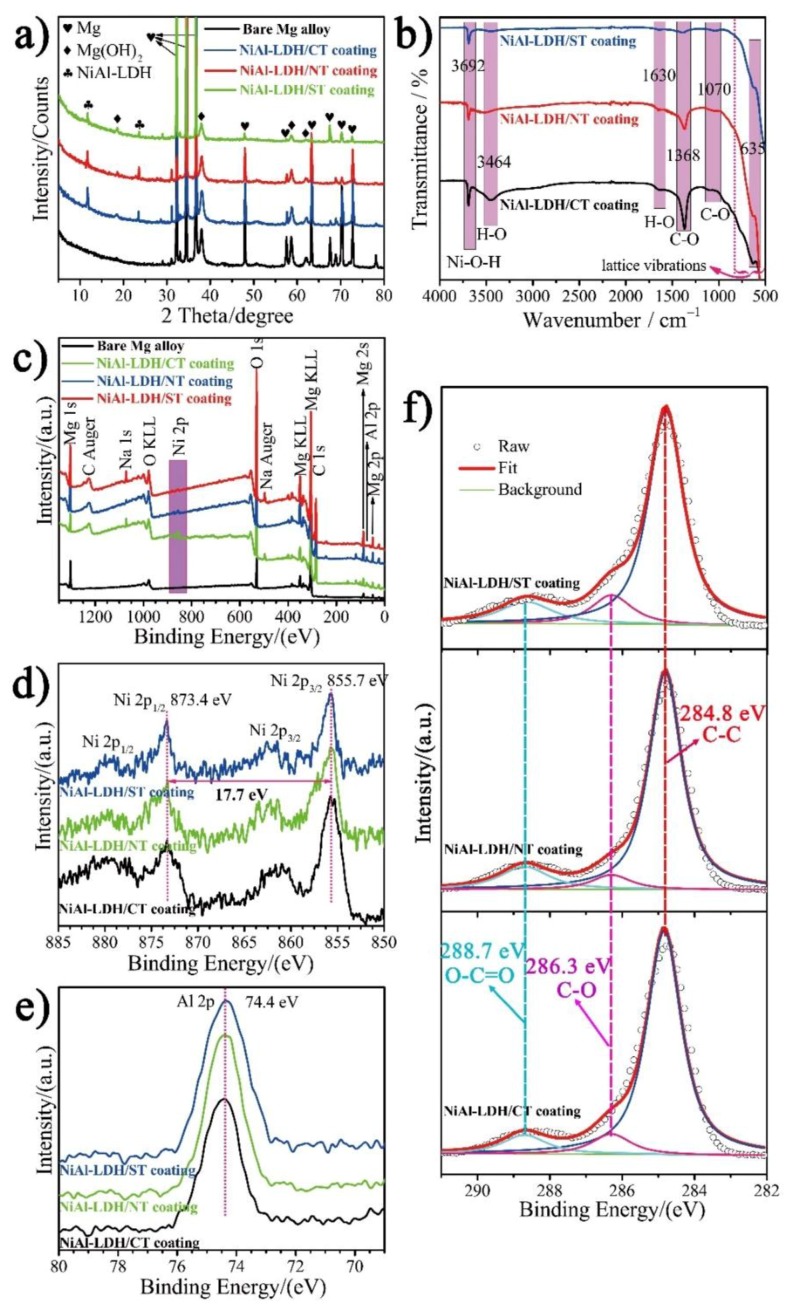
(**a**) XRD patterns; (**b**) FT-IR; and (**c**–**f**) XPS spectra of different NiAl-LDH coatings.

Figure 3a shows the XRD patterns of Mg alloy and NiAl-LDH coatings obtained with different nickel salts. For the substrate, three strong peaks at (2θ) 32.20°, 34.40°, and 36.62°, and several relatively weak peaks at 47.83°, 63.06°, and 72.50°, etc. are ascribed to the characteristic diffraction peaks of Mg (PDF 35-0821). These small and weak peaks at 18.59°, 38.02°, 58.64°, and 62.07° are the characteristic peaks of Mg(OH)_2_ (PDF 07-0239) [19,20]. The XRD patterns of all coatings are almost identical and two new peaks appear at ca. 11.73° and 23.58°, which correspond to the (003) and (006) planes of NiAl-LDH (PDF 15-0087) [15]. Figure 3b shows that all samples present almost the same absorption peaks in the FT-IR spectra. The relatively strong peak at 3692 cm^−1^ is related to the O–H stretching vibration of Ni–O–H, and the broadening adsorption peaks at 3464 cm^−1^ and 1630 cm^−1^ are associated with the O–H stretching and bending vibrations from interlamellar water molecules [21] owing to formation of hydrogen-bond. The absorption bands located at 1368 and ca. 1070 cm^−1^ correspond to the asymmetrical and symmetrical stretching vibrations of C–O in CO_3_^2−^, respectively. The weak bands below 800 cm^−1^ are assigned to lattice vibrations of metal-oxygen (M–O) in the brucite-like layers [8]. XPS survey spectrum in Figure 3c shows that the primary elements of substrate are Mg and O, and C 1s peaks is hardly seen. After coating, two new peaks appeared at ca. 856.5 and 74.2 eV which are assigned to Ni 2p and Al 2s, respectively. In addition, significant intensification of C 1s peak at ca. 284 eV is observed. The Ni 2p high-resolution XPS spectra exhibit two major peaks along with two pairs of shake-up satellites (Figure 3d). The major peaks at 873.4 and 855.7 eV relating to Ni 2p_1/2_ and Ni 2p_3/2_ with a spin-energy separation of 17.7 eV are characteristics of Ni (II) in Ni(OH)_2_. The Al 2p spectra showing peaks at ca. 74.4 eV (Figure 3e) are ascribed to Al^3+^ species (Al–O). The C 1s spectrum is deconvoluted into three separate peaks related to different types of carbon bonds including C–C at 284.8 eV owing to adventitious carbon, C–O (286.3 eV) and O–C=O (288.7 eV) from CO_3_^2−^ (Figure 3f). These characterizations demonstrate the successful formation of NiAl-LDH crystal phase on the matrix.

**Figure 4:**
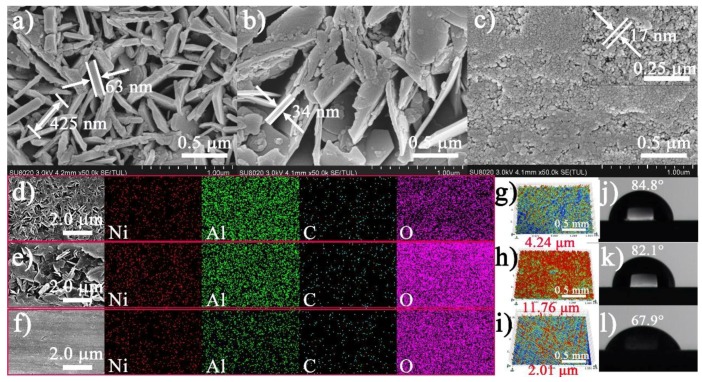
(**a**–**c**) SEM; (**d**–**f**) SEM-EDS mapping; and (**g**–**i**) 3D roughness images of the surface morphologies; and (**j**–**l**) water contact angles for (**a**,**d**,**g**,**j**) NiAl-LDH/CT, (**b**,**e**,**h**,**k**) NiAl-LDH/NT, and (**c**,**f**,**i**,**l**) NiAl-LDH/ST coatings.

Figure 4a–c exhibits the matrix surface fully covered with vertically aligned nanoflake arrays for the NiAl-LDH/CT coating, both vertically and horizontally aligned, and randomly-inclined nanoflakes for the NiAl-LDH/NT coating, and nanonodules for the NiAl-LDH/ST coatings. These coatings are composed of Ni, Al, C, and O, and distribute uniformly all over the LDHs coating surfaces (Figure 4d–f). The thickness and side length of the nanoflakes are 63 and 425 nm, respectively, for NiAl-LDH/CT coating. The thickness of the nanoflakes for NiAl-LDH/NT coating is about 34 nm, and the length cannot be determined due to random orientation and irregular shape which result in a much higher *R*_a_, 11.76 μm, than that of NiAl-LDH/CT coating (4.24 μm) (Figure 4g–i). The NiAl-LDH/ST coating exhibits a flat surface packed by uniform nanonodules with a size of ca. 17 nm, producing the lowest *R*_a_, 2.01 μm, among the three types of NiAl-LDH coatings. The different shapes of the deposits result in different water contact angles (Figure 4j–l). The water contact angles of NiAl-LDH/CT and NiAl-LDH/NT coatings are 84.8° and 82.1°, respectively, while the value of NiAl-LDH/ST coating is only 67.9°. The variation in water contact angles led to a slight difference in corrosion inhibition for these coatings.

**Figure 5:**
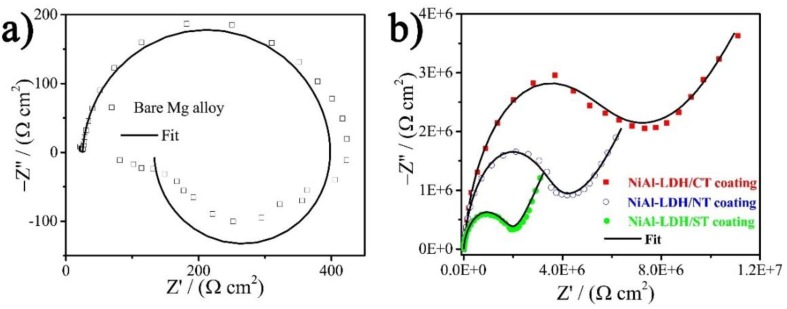
Nyquist plots of (**a**) bare Mg alloy and (**b**) different coatings.

**Figure 6:**
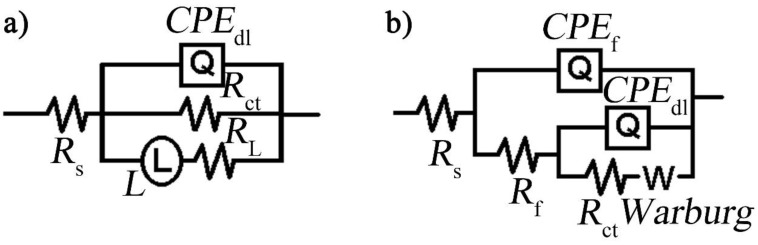
The equivalent circuit models used for fitting the EIS results of the (**a**) bare Mg alloy and (**b**) different NiAl-LDH coatings. The equivalent circuit (EC) model of the Mg alloy for fitting is *R*_s_(*Q*_dl_*R*_ct_(*R*_L_L)) where *R*_s_ is solution resistance, *Q*_dl_ is double layer capacitance, *R*_ct_ is charge transfer resistance, *R*_L_ is inductance resistance, and L is inductance. The EC model for coatings is *R*_s_(*Q*_f_(*R*_f_(*Q*_dl_(*R*_ct_*Z*_w_)))) where *Q*_f_ and *R*_f_ are capacitance and resistance of the coatings, respectively. *Z*_w_ is Warburg diffusion resistance.

Electrochemical impedance spectroscopy (EIS) was carried out in 3.5 wt % NaCl corrosive electrolyte to evaluate the corrosion resistance of the three NiAl-LDH coatings [22], which is depicted in Figure 5. EIS results show that Mg alloy has a two-time constant (Figure 5a), i.e., one capacitive loop at high frequency and one inductive loop with ranges from intermediate to low frequency region, which are ascribed to the electric double layer at the electrode/electrolyte interface and relaxation diffusion of corrosion products such as Mg(OH)^+^_ads_, respectively [20,23]. According to the fitting results by use of an equivalent circuit (EC) model as given in Figure 6a [20], the charge transfer resistance (*R*_ct_) of the bare Mg alloy is only 369.20 Ω cm^2^. After deposition of NiAl-LDH films, totally different Nyquist plots were observed. The fitting results based on an EC model consisting of *R*_f_, *R*_ct_, and *Z_w_* (Figure 6b) are listed in Table 2. All the coatings show a very high *R*_f_ along with a remarkable increment of *R*_ct_, manifesting the significant enhancement of corrosion protection by the coatings. In contrast, NiAl-LDH/CT coating exhibits the highest *R*_f_ (6.2 MΩ cm^2^) and *R*_ct_ (3.5 MΩ cm^2^), followed by NiAl-LDH/NT coating with medium *R*_f_ (3.7 MΩ cm^2^) and *R*_ct_ (1.8 MΩ cm^2^), and the NiAl-LDH/ST coating with the lowest *R*_f_ (1.4 MΩ cm^2^) and *R*_ct_ (0.72 MΩ cm^2^). The impedance modulus at a low frequency, such as |*Z*|*_f_*_= 0.1 Hz_, which can be obtained directly from the Bode plots without fitting, also represent the corrosion-resistant capability of a coating [20,21]. It can be seen from Table 2 that the NiAl-LDH/CT coating also possesses the highest |*Z*|*_f_*_= 0.1 Hz_, 11.6 MΩ cm^2^, in comparison with that values of NiAl-LDH/NT (6.7 MΩ cm^2^) and NiAl-LDH/ST (3.4 MΩ cm^2^) coatings. The biggest radius of curvature in Figure 5b also confirms the best corrosion resistance of NiAl-LDH/CT coating.

**Figure 7:**
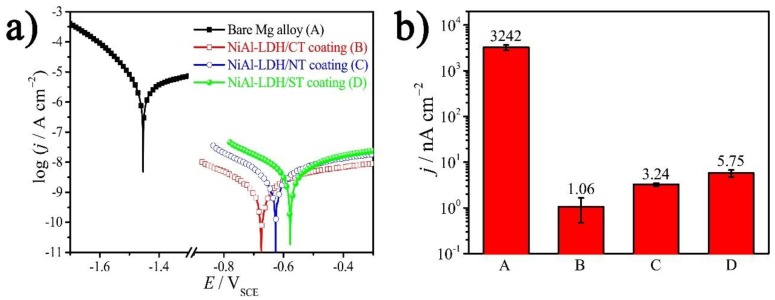
(**a**) Tafel curves of the bare Mg alloy and different NiAl-LDH coatings in NaCl corrosive medium. (**b**) The corresponding *j*_corr_ of substrate and coatings extracted from the Tafel curve.

A further Tafel test was performed to ascertain the corrosion potential, *E*_corr_ vs. SCE, and *j*_corr_ of the samples [24,25], the results of which are shown in Figure 7 and listed in Table 3. The *E*_corr_ and *j*_corr_ of a bare Mg alloy are −1450 mV and 3242 nA cm^−2^, respectively. After deposition of NiAl-LDH/CT film, the *E*_corr_ shifts positively by ca. 800 mV to −674 mV, and *j*_corr_ is decreased by a factor of 3058 in comparision with that value of the matrix.The *j*_corr_ of NiAl-LDH/NT and NiAl-LDH/ST coatings are 3.24 and 5.75 nA cm^−2^, respectively, which are in positive agreement with the variation of |*Z*|*_f_*_= 0.1 Hz_ because a coating with higher impedance always has lower *j*_corr_ and better corrosion resistance. However, it is worth noting that a material with a lower *j*_corr_ does not always possess a higher *E*_corr_. For example, the NiAl-LDH/ST coating has the highest *j*_corr_ but the lowest *E*_corr_ (Table 3). That is because *E*_corr_ is a thermodynamic parameter, which stands for the corrosion tendency, while corrosion rate is proportional to the *j*_corr_, i.e., there is no direct relationship between *E*_corr_ and *j*_corr_.

The high capacity and slight difference in corrosion resistance of the three different NiAl-LDH coatings are ascribed to the high affinity of LDHs towards CO_3_^2−^ in comparison with the corrosive Cl^−^, the different orientations of the nanosheets, and the different shapes of the deposition, which is illustrated in Figure 1. The corrosive species cannot be exchanged with the brucite-like layer and have to exchange with the CO_3_^2−^ anions to arrive at the substrate surface, but the ion-exchange is not easy due to the high affinity of LDHs to CO_3_^2−^ anions, enabling high enhancement in corrosion inhibition in all the as-prepared NiAl-LDH coatings. Due to the vertically-aligned nanosheets in the NiAl-LDH/CT coating, the carbonate anions distribute evenly in the interlayer spaces, which further increase the difficulty of the ion-exchange between CO_3_^2−^ anions and corrosive species (Case I in Figure 1). For the NiAl-LDH/NT coating, the basal spacing becomes larger at some locations and CO_3_^2−^ anions distribute unevenly in the interlayer spaces owing to irregular orientation of the nanosheets, which increases the probability for corrosive species to arrive at the substrate surface by going through fewer CO_3_^2−^ anions, resulting in reduction of corrosion inhibition (Case II in Figure 1). In general, the dense plate-like arrangement of LDHs is favorable for increasing corrosion resistance of the film due to lower exposure of CO_3_^2−^ anions for ion-exchange. However, for the NiAl-LDH/ST coating in this work, nanomodules with many obvious voids rather than nanoflakes were formed, as demonstrated by the SEM image in Figure 4c, which decreases the corrosion-resistant capability of the LDHs film. In addition, the smaller water contact angle of NiAl-LDH/ST coating in comparison with that of NiAl-LDH/CT and NiAl-LDH/NT coatings should also be responsible for the decreasing corrosion inhibition of NiAl-LDH/ST coating. It is worth noting that although these explanations above account for the difference in corrosion resistance of the three different coatings, the influences of the different orientation and shapes of the deposition on the corrosion inhibition are not significant because all the *j*_corr_ of the coatings remain in the level of nA cm^−2^.

## 4. Conclusions

A facile hydrothermal strategy was progressed to achieve in-situ NiAl-LDH coatings on Mg alloy to improve the corrosion protection. All the NiAl-LDH coatings obtained by different nickel salts show remarkable enhancement in corrosion inhibition in NaCl solution compared with Mg alloy substrate, which is attributed to the strongly affinity between charge-compensating CO_3_^2−^ and brucite-like layers. The different orientation of the nanosheets and the different shapes of the deposition are mainly responsible for the slight difference in corrosion inhibition performance among the three different coatings. The NiAl-LDH/CT coating deposited by carbonate shows relatively the highest |*Z*|*_f_*_= 0.1 Hz_ and lowest *j*_corr_, suggesting its best corrosion inhibition. It is believed that these findings may inspire the design and development of other LDH nanosheet arrays, such as NiCr-LDH arrays, as highly enhanced corrosion protection film for a susceptible lightweight metal matrix.

## Figures and Tables

**Table 1:** Corrosion inhibition of different layered double hydroxide (LDH) coatings on Mg alloy from most recent published literature.

Substrate	LDH Coatings	Corrosive Medium	*E*_corr_ V vs. SCE	*j*_corr_ (A cm^−2^)	Ref.
Mg alloy	MgAl-NO_3_^−^	Phosphate buffer saline	−1.53	3.63 × 10^−7^	[14]
Anodized AZ31 Mg alloy	MgAl-NO_3_^−^	3.5 wt % NaCl solution	−0.47	9.48 × 10^−7^	[8]
Anodized AZ31 Mg alloy	MgAl-VO_3_^−^	3.5 wt % NaCl solution	−0.40	2.48 × 10^−7^	[8]
Anodized AZ31 Mg alloy	MgAl-NO_3_^−^	3.5 wt % NaCl solution	−1.34	1.18 × 10^−7^	[10]
AZ31 Mg alloy	MgAl-5-fluorouracil	Phosphate buffer saline	−1.12	3.34 × 10^−5^	[13]
Plasma electrolytic oxidation pretreated AZ31 Mg alloy	MgAl-5-fluorouracil	Phosphate buffer saline	−1.20	3.92 × 10^−6^	[13]
AZ31 Mg alloy	MgAl-5-fluorouracil	Phosphate buffer saline	−1.42	3.27 × 10^−5^	[16]
Anodized AZ31 Mg alloy	MgFe-NO_3_^−^	3.5 wt % NaCl solution	−1.44	1.09 × 10^−6^	[10]
Anodized AZ31 Mg alloy	MgCr-NO_3_^−^	3.5 wt % NaCl solution	−1.47	2.16 × 10^−6^	[10]
AZ91D Mg alloy	ZnAl-VO_3_^−^	3.5 wt % NaCl solution	−1.30	2.21 × 10^−6^	[17]
AZ91D Mg alloy	ZnAl-Cl^−^	3.5 wt % NaCl solution	−1.39	2.52 × 10^−6^	[17]
AZ91D Mg alloy	ZnAl-NO_3_^−^	3.5 wt % NaCl solution	−1.42	1.33 × 10^−5^	[17]
AZ31 Mg alloy	MgAl-CO_3_^2−^	3.5 wt % NaCl solution	−0.36	8.40 × 10^−7^	[18]
Anodized AZ31	MgAl-CO_3_^2−^	3.5 wt % NaCl solution	−0.29	3.50 × 10^−7^	[18]

**Table 2:** Parameters of EIS for three different NiAl-LDH coatings.

Samples	*Q*_f_/10^−9^ (S s^n^ cm^−2^)	*R*_f_ (MΩ cm^2^)	*Q*_dl_/10^−9^ (S s^n^ cm^−2^)	*R*_ct_ (MΩ cm^2^)	*W*/10^−7^ (S s^0.5^ cm^−2^)	|Z|*_f_*_= 0.1 Hz_ (MΩ cm^2^)	χ^2^/10^−3^
NiAl-LDH/CT coating	2.1 ± 0.029	6.2 ± 0.17	62.8 ± 7.6	3.5 ± 0.45	3.5 ± 0.17	11.6	0.31
NiAl-LDH/NT coating	1.5 ± 0.021	3.7 ± 0.14	113.5 ± 9.8	1.8 ± 0.22	3.5 ± 0.35	6.7	0.22
NiAl-LDH/ST coating	0.84 ± 0.087	1.4 ± 0.15	24.5 ± 2.6	0.72 ± 0.076	6.9 ± 0.27	3.4	1.5

**Table 3:** Parameters of Tafel curves for bare Mg alloy and different NiAl-LDH coatings.

Samples	*E*_corr_ (mV)	*j*_corr_ (nA cm^−2^)	*β*_a_ (mV dec^−1^)	−*β*_c_ (mV dec^−1^)
Bare Mg alloy	−1450 ± 52	3242 ± 425	331	95
NiAl-LDH/CT coating	−674 ± 42	1.06 ± 0.59	190	171
NiAl-LDH/NT coating	−625 ± 96	3.24 ± 0.25	244	184
NiAl-LDH/ST coating	−577 ± 51	5.75 ± 1.03	270	188
